# Evaluating patient participation in value‐based healthcare: Current state and lessons learned

**DOI:** 10.1111/hex.13945

**Published:** 2024-01-18

**Authors:** Henrike J. Westerink, Mirjam M. Garvelink, Cornelia F. van Uden‐Kraan, Ouisam Zouitni, Hans A. J. Bart, Philip J. van der Wees, Paul B. van der Nat, the Santeon Patient Participation Study Group

**Affiliations:** ^1^ Department of Value Improvement St. Antonius Hospital Nieuwegein The Netherlands; ^2^ Scientific Center for Quality of Healthcare (IQ healthcare) Radboud University Medical Center Nijmegen The Netherlands; ^3^ Santeon Utrecht The Netherlands; ^4^ Client Council St. Antonius Hospital Utrecht/Nieuwegein The Netherlands

**Keywords:** mixed methods, patient involvement, Patient and Public Evaluation Tool (PPEET), value‐based healthcare

## Abstract

**Introduction:**

Value‐based healthcare (VBHC) focusses on increasing value for patients. Hospitals aim to implement VBHC via value improvement (VI) teams for medical conditions. To determine the patient's perspective on value, collective patient participation is important in these teams. We therefore evaluated the current state of patient participation in VI teams and share lessons learned.

**Methods:**

This mixed‐methods study was conducted at seven collaborating hospitals in the Netherlands. A questionnaire (the public and patient engagement evaluation tool) was tailored to the study's context, completed by VI team members (*n* = 147 from 76 different VI teams) and analysed with descriptive statistics. In addition, 30 semistructured interviews were held with VI team members and analysed through thematic analysis. Data were collected between February 2022 and January 2023 and were triangulated by mapping the quantitative results to the interview themes.

**Results:**

Thirty‐eight of the 76 included VI teams reported using a form of patient participation. Many respondents (71%) indicated a lack of a clear strategy and goal for patient participation. Multiple VI team members believed that specific knowledge and skills are required for patients to participate in a VI team, but this led to concerns regarding the representativeness of participating patients. Furthermore, while patients indicated that they experienced some level of hierarchy, they also stated that they did not feel restricted hereby. Lastly, patients were satisfied with their participation and felt like equal VI team members (100%), but they did mention a lack of feedback from the VI team on their input.

**Conclusion:**

The results imply the lack of full implementation of patient participation within VI teams. Guidelines should be developed that provide information on how to include a representative group of patients, which methods to use, how to evaluate the impact of patient participation, and how to give feedback to participating patients.

**Patient and Public Contribution:**

Two patient advisors were part of the research team and attended the research team meetings. They were involved as research partners in all phases of the study, including drafting the protocol (e.g., drafting interview guides and selecting the measurement instrument), interpreting the results and writing this article.

## 1 INTRODUCTION

In the last decade, the concept of value‐based healthcare (VBHC) has become a prominent force in healthcare and has been embraced by many countries across the world to improve quality of care and counter rising healthcare costs.^1^ VBHC can be defined as a way to organize care that focusses on increasing value for those being cared for, that is, the patients. Within VBHC, value has been defined as patient‐relevant outcomes relative to the costs needed to achieve these outcomes.^2^ Different strategies to implement VBHC can be found, and an often adopted strategy is to set up multidisciplinary value improvement (VI) teams that are responsible for VIs in the care for a specific medical condition.^1^, ^3^ Patient participation within these VI teams is of great importance to facilitate the incorporation of their perspectives on value.^4^

Patient participation can occur on two distinct levels,^5^ and with different intensities.^6^ At the individual level, patient participation is about the individual patient's care, for example, in shared decision‐making or self‐management.^7^, ^8^ At the collective level, patient participation is defined as actively involving the patient in organization‐wide or (inter)national projects, where the outcome of the project has an effect beyond their individual care.^5^, ^9^ Patient participation in multidisciplinary VI teams is an example of patient participation at the collective level.^10^ Within these VI teams, patients can contribute to identifying and evaluating patient‐relevant outcomes and improvement initiatives.^11^, ^12^

The focus of literature regarding patient participation in VBHC has mainly been at the individual level, that is, by using outcome data for shared decision‐making.^13^, ^14^ However, there is a lack of evidence and examples of best practices for collective patient participation,^15^ and multiple barriers to participation have been mentioned in literature.^16^ For example, a frequently mentioned barrier is that generally higher educated patients are involved at the collective level, resulting in underrepresentation of a large group of (lower educated) patients at this level.^17^ These barriers and lack of best practices for collective patient participation often result in tokenistic patient participation,^18^ which occurs when patients participate but have little to no influence.

Although the impact of collective patient participation in VBHC has been stipulated,^4^, ^11^, ^12^ thus far, there is a paucity of literature on practical implementation and evaluation of collective patient participation within VBHC.^15^ Therefore, the process and impact of collective patient participation need to be evaluated to identify best practices for patient participation in VBHC. In this study, we aimed to evaluate the experiences with collective patient participation within multidisciplinary VI teams using a mixed‐methods approach.

## 2 METHODS

### 2.1 Context

We conducted a multicentre study between February 2022 and January 2023 in a consortium of seven Dutch hospitals (Santeon hospitals) that collaborate in implementing VBHC by actively measuring, benchmarking and improving the value of care for 15 medical conditions (e.g., breast cancer, chronic kidney disease and stroke). Multidisciplinary VI teams per medical condition have been set up in each of the seven hospitals, with a few exceptions when the hospital does not provide care for that condition. These VI teams consist of healthcare professionals, project leaders, managers and data analysts who work together to achieve VIs. For each of the 14 conditions, outcome, process and cost data are being collected and compared between the seven hospitals. The VI teams then select improvement initiatives based on the insights from the (comparison of) data. Detailed information on the method of the Santeon VI teams can be found in the articles by Daniels et al. and Engels et al.^12^, ^19^ Since the start of these VI teams, Santeon has strived to include at least one patient in every VI team, but this has been experienced as challenging. Therefore, we aimed to evaluate collective patient participation within the Santeon VI teams to identify the lessons learned.

### 2.2 Study design

**Figure 1:**
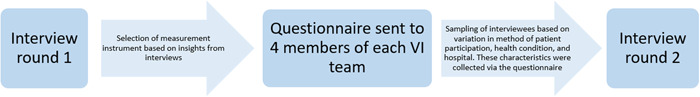
Schematic overview of data collection.

This study had a mixed‐methods design and was based on a case study methodology consisting of questionnaires and two rounds of interviews with VI team members. The first round of interviews was conducted to select an appropriate questionnaire based on the experiences of VI team members. Subsequently, this questionnaire was completed by the VI team members. The second round of interviews was conducted to deepen the results of the questionnaire. Questionnaire data were triangulated with interview data to gain in‐depth insight into the current state of patient participation in the VI teams. See Figure 1 for a schematic overview of the data collection.

### 2.3 Data collection

#### 2.3.1 Interviews with VI team members

Qualitative data collection and analysis are reported according to the COREQ guidelines.^20^ Different VI team members were interviewed in‐depth about patient participation within their team in two rounds of semistructured interviews. We purposively sampled VI team members to be interviewed based on variations in medical conditions, their role within the VI team (e.g., medical specialist, nurse, support staff and patient), hospital location and in the second round of interviews also on the method of patient participation within their VI team (identified with the questionnaire). Different VI team members were approached for the two rounds. Three different semistructured interview guides were used for the two interview rounds (Supporting Information S1: Appendix A), where in round one we used a different interview guide for the patients and staff members. A total of 43 VI team members were invited via email. They had the choice to do the interview face‐to‐face or via videoconferencing. The interviews were audio‐recorded and field notes were made during the interviews. Interviews were performed until data saturation was reached.

#### 2.3.2 Questionnaire

Based on the first round of interviews, the research team selected a questionnaire from the list of measurement instruments of the Engagement Assessment Toolkit for patients and the public (https://ceppp.ca/en/evaluation-toolkit/)^21^ to evaluate patient participation in VI teams. During a meeting with the research team, all measurement instruments from the Engagement Assessment Toolkit were discussed and the domains of the instruments were compared to preliminary findings of the interviews. Based on this discussion, the Patient Participation Engagement Evaluation Tool (PPEET) was selected to be best fitting to the context of VI teams.^22^ This questionnaire addresses subjects that were also discussed during the first round of interviews. The PPEET is a validated questionnaire and consists of separate questionnaires for patients and staff, each consisting of three modules. Questions of the PPEET can be answered based on a 5‐point Likert scale (totally disagree to totally agree) or with yes/no. The Dutch version of the PPEET was used in this study.^23^

We selected module B as the main questionnaire, which focuses on ongoing engagement activities. We have added a few questions to both the patient and the staff questionnaire (see Supporting Information S2: Appendix B):

1. *Patient questionnaire*: We added four self‐formulated questions (questions 4, 9, 10 and 14) to tailor the PPEET to the context of the VI teams based on preliminary findings of the first round of interviews.
2. *Staff questionnaire*: We added three questions from module A (focusing on the planning of patient participation) and two from module C (focusing on the impact of patient participation). Furthermore, we added one self‐formulated question to the PPEET based on the preliminary findings of the first round of interviews.

The questionnaire was pilot‐tested with ten VI team members. Respondents were only asked to answer relevant questions, so if a respondent indicated not having used any form of patient participation, they were not asked any more detailed questions on their experiences with patient participation. Respondents were required to answer all questions that they received to make sure that we did not have any missing data.

The questionnaire was sent out by the project leaders of the VI teams via a data management system (REDCap) to four members of each VI team, that is, a patient and three staff members (e.g., a medical specialist, a nurse and someone with a supporting role such as a project leader or data analyst). Reminder emails were sent after two and four weeks.

### 2.4 Data analysis

The semistructured interviews of both rounds were recorded and transcribed verbatim using transcription software (Amberscript). The transcripts were then checked and corrected by the first author (H.J.W.), and member‐checked by the interviewees. Data were coded with Atlas.ti using open coding with an inductive approach to let themes arise from the data. To increase reliability, all interviews were double‐coded by a member of the research team (O.W.). Agreement between the coding was checked after coding every five interviews, and any discrepancies were resolved by consensus. The two researchers (H.J.W. and O.W.) analysed the codes through thematic analysis and discussed the identified themes until a consensus was reached.

For the Likert‐type questionnaire data, the percentages of respondents who (totally) agreed with the individual questions (score four or five on the Likert scale) of the measurement instrument were calculated. For the yes/no questions, the percentages of respondents that answered with ‘yes’ were calculated per question. Data from the pilot test of the questionnaire was also included in the analysis.

#### 2.4.1 Triangulation

The quantitative data were then triangulated (convergence triangulation) with the qualitative data by mapping the questions of the PPEET to the interview themes. Agreements and discrepancies between the quantitative and qualitative data were discussed in the results. A step‐by‐step guide to the data analysis can be found in Supporting Information S3: Appendix C.

#### 2.4.2 Trustworthiness

We strived for credible results by continuing data collection until data saturation was reached, discussing identified themes with all co‐authors and using data triangulation of the quantitative and qualitative results. Furthermore, participants checked checked the transcripts and preliminary results. All interviews were conducted by H.J.W., who had previous experience and training in conducting and analysing interviews. Interviewees had no prior relationship with the interviewer. To increase the transferability of the results, the data triangulation was discussed with all co‐authors and consensus was reached on the findings of the study. Lastly, confirmability was accounted for by double‐coding the interview data.

## 3 RESULTS

### 3.1 Participant characteristics

We interviewed a total of 30 VI team members from 25 different VI teams, of which 12 were patients (response rate = 70%). Table 1 shows the participant characteristics. VI team members who refused to participate in the study mainly mentioned time constraints as a reason for their refusal to participate. Data saturation was reached after 22 interviews, but we continued with the other eight interviews since these were already planned. The interviews had an average duration of 26 (±7) min, starting from the first interview question.

**Table 1:** Participant characteristics.

Characteristics	*N* interviews round 1	*N* questionnaire	*N* interviews round 2
Total	15	147	15
Role			
Patient	6	15	6
Medical specialist	2	39	2
Nurse	1	36	1
Support staff	4	57	4
Other	2		2
Medical condition			
Birth care	2	14	1
Breast cancer	3	16	2
Chronic kidney disease	2	7	
Colon cancer	1	11	1
Coronary artery disease		6	
Cerebrovascular accident		14	4
Diabetes	2	14	2
Hip fracture		8	
Hip osteoarthritis	1	6	
Inflammatory bowel disease	3	14	
Knee osteoarthritis		7	2
Lung cancer		9	1
Prostate cancer	1	9	
Rheumatoid arthritis		5	2
Vulnerable elderly		7	

### 3.2 Patient participation within VI teams

The questionnaire was completed by 147 VI team members from 76 different VI teams for 15 medical conditions. Of these 76 VI teams, 24 had a patient in their team, 14 used another method, and 18 had no patient participation in their team. In the remaining 20 teams, it was unclear which method was used for patient participation, as respondents of the same team gave conflicting answers. All of the patients who completed the questionnaire were highly educated (university or university of applied sciences degree) and 47% had a full‐time job. Most patients were older than 51 years old (67%).

### 3.3 Triangulated interview and questionnaire results

A total of nine different themes were identified from the interviews. Table 2 shows the triangulated results of the PPEET questionnaire results mapped to the interview themes including interview quotes. Overall, we mainly discuss results from teams with (some) experience in patient participation, as these results provide better insight into the lessons learned compared to VI teams, where they have not used any form of patient participation. All quantitative results are presented in percentages.

**Table 2:** Evaluation of patient participation in value improvement teams based on qualitative (interview) and quantitative (PPEET questionnaire) data.

Theme	Interview subthemes	Example quote from the interview	% of participants who (totally) agree with PPEET^24^ question
1. Goals and motivation for PP	1.1. Motivation of patient 1.2. Priority of PP 1.3. Role patient/staff	1.1: ‘I feel like I can give something back, maybe help other patients … and also the hospital’ (Patient 1)	Staff: There was a clear statement of the objectives for the engagement: 39% Staff: The goals for the engagement component were shared with participants: 75%
2. Strategy for PP	2.1. PP as a subject of discussion 2.2. Strategy for PP 2.3. Responsibility for PP 2.4. Patient selection for the VI team 2.5. PP methods	2.3: ‘I have always been an assertive patient … so maybe that's why they asked me if I wanted to join [the VI team]’ (Patient 2) 2.3: ‘I think that the healthcare professionals should be responsible [for patient participation]. Because when you want to improve the care for a specific patient group, then the experts that treat that group should be in the lead’ (Staff 1) 2.5: ‘I would really like to have more input from patients. But the method and how to do it, is what I'm struggling with’ (Staff 2)	Staff: The VI team has a clear strategy to recruit those most affected by the outputs of this project (e.g., relevant lived experience, sociodemographic or geographic communities): 29% Staff: The VI team has a clear strategy to actively engage patients: 31% Staff: We worked with other (patient) organizations as part of the engagement component of this project: 27% yes Staff: We were able to identify shared goals with our partners through this process: 68% Staff: Do you plan to collaborate with these partners again in the future?: 92% yes
3. Context of VI team influences PP	3.1. VI team characteristics 3.2. Process of VI meetings 3.3. Content discussed in the VI team	3.2: ‘I think that everyone is positive towards patient participation. That is due to the medical lead. She is the ambassador and she motivated the rest’ (Patient 3)	NA
4. Resources required for PP	4.1. Resources of the team for PP 4.2. Compensation for patients	4.2: ‘I think that it [patient participation] is a time issue. It can slow down the process when the input [of patients] has to be collected first, and therefore they might be a little bit sceptical of the added value’ (Staff 3)	Staff: The financial, logistical and information needs of participants (e.g., travel, dietary, interpretive, childcare, etc.) were accommodated: 38% Staff: Adequate time was allocated to plan and implement the engagement component: 46% Pt: The supports I need to participate in the VI team are available (e.g., travel, childcare): 89%
5. Knowledge and skills in PP required from patients and staff	5.1. Required skills of patients 5.2. Previous experience of patients with PP 5.3. Characteristics of patient 5.4. Knowledge of patient	5.2: ‘They [the staff] knew that I was already involved in “patient partner” projects and then they asked me if I wanted to do this [being a VI member]’ (Patient 4)	Staff: I would like to participate in a training on patient participation: 44% yes Pt: I have a clear understanding of the purpose of the VI team: 100% Pt: I have enough information to be able to carry out my role: 100%
6. Representativeness as a barrier for PP	6.1. Representativeness of patients 6.2. Number of patients in the VI team	6.1: ‘You will always hear from 1 or 2 highly educated, interested, verbally strong, generally positive patients, otherwise they would not be here. So you get very selective input’ (Staff 4)	Staff: The perspectives of those who will be most affected by the outputs of this project were reflected through those who participated in the engagement: 74% Pt: I feel like I can represent the patient's perspective: 100% Pt: I feel more comfortable because I am not the only patient in the VI team: 56% Pt: The individuals participating in the VI team represent a broad range of perspectives: 87% Pt: A wide range of views on discussion topics is shared: 80%
7. Relationship among VI team members influences PP	7.1. Relationship among VI team members 7.2. Hierarchy in VI team	7.2: ‘You do notice that the hierarchy, which is common in the hospital, is also present there [in the VI team]’ (Patient 1)	Staff: I can speak freely with the patient at the table: 80% Pt: I am able to express my view freely: 100% Pt: I feel like an equal VI team member: 100%
8. Feedback and evaluation is important in PP	8.1. Feedback on input 8.2. Evaluation of PP 8.3. Patient views on PP 8.4. Staff views on PP 8.5. Improvements should be made	8.1: ‘Sometimes I have the feeling that I'm not sure whether they took the input [given by patient] into account’ (Patient 6)	Staff: Participants were told how the input from the engagement component would be used by the organization: 81% Staff: Overall, I was satisfied with the engagement component of this project: 41% Pt: I get sufficient feedback on how my input is used: 73% Pt: Overall, I am satisfied with the patient participation in this project: 87% Pt: This initiative for patient participation is a good use of my time: 87%
9. Impact of PP	9.1. Added value of PP 9.2. Improvement in PP 9.3. Burden for patient Impact PP	9.3: ‘When you [the patient] are in a crisis [with your health], then you have to be occupied with that, and not with the process and what the hospital is asking from you’ (Staff 5)	Staff: The output generated from the engagement component associated with this project influenced the project's outcome: 51% Staff: The output generated from the engagement component associated with this project was taken seriously by those in a position to act on it: 78% Staff: The engagement component added value to the project it supported: 90% Staff: As a result of my involvement in the engagement component associated with this project, I will be comfortable leading future engagement activities: 60% Pt: I feel that my views are heard: 93% Pt: I am confident that the VI team takes my provided feedback into consideration: 93% Pt: I think that the work of the VI team makes a difference to the work of the hospital: 87% Pt: I think that the hospital is achieving its stated objectives for the VI team: 67% Pt: As a result of my participation in the VI team, I am better informed about my medical condition and/or the healthcare system: 73%

Abbreviations: NA, not available; PP, patient participation; PPEET, PPEET, Public and Patient Engagement Evaluation Tool^24^; pt, patient; VI, value improvement.

#### 3.3.1 Goals and motivation for patient participation

Patients mentioned several reasons why they were motivated to participate in the VI team: to return something, to improve care or because they were personally interested in the topics discussed in the VI team meetings. Staff members mentioned that they wanted to let patients participate because they would like to gain more insight into the patients' perspectives on care to identify or evaluate improvement initiatives. In the first interview round, a few staff members indicated that they did not have a patient in their team since they believed that it was their role to represent the patient perspective and that no actual patient participation was therefore needed within the VI team.

Furthermore, according to multiple staff members, patient participation had been a low priority for their VI team and was often top‐down organized. Only 39% of the staff members indicated that their VI team had a clear statement regarding the objectives of patient participation. In addition, several patients mentioned that they were not aware of the exact goal for their participation or their role in the VI team, while 75% of the staff members of teams in which patients participated indicated that the goals for patient participation were shared with the patients.

#### 3.3.2 Strategy for patient participation

Overall, staff members believed that patient participation should be discussed more often during their VI meetings. Furthermore, staff members indicated a lack of strategy for patient participation, and they expressed the need for a guideline on patient participation within VI teams. In addition, they mentioned that no one was specifically responsible for patient participation within the VI team. Different staff members were suggested that could be responsible, such as the project leader, medical lead or marketing department. The lack of strategy was also identified in the questionnaire, where only 31% of the staff had a clear strategy for patient participation.

Moreover, a lack of strategy for the recruitment of patients for the VI team was mentioned. Staff members purposively selected patients based on whether they believe that they are able to participate in the VI team based on their knowledge and skills (e.g., in communication). Quantitative data showed that 29% of the staff indicated having a clear strategy specifically for the recruitment of patients for the VI team.

Staff members indicated that the method of patient participation should be different for different medical conditions and subjects that are discussed. Other possible methods of patient participation mentioned by staff members included using aggregated PROMs and PREMs data, using a patient panel, sending out questionnaires, conducting interviews, and conducting focus groups. Each of these methods was believed to provide better insight into the perspectives of multiple patients and was therefore seen as more representative than one or two patients in the VI team. However, also downsides of these methods were mentioned, such as having low response rates to questionnaires, or being top‐down because the staff members decide what will be asked in the questionnaire or interview. Furthermore, staff members expressed a wish to have more contact with patient organisations to gain insight into the perspective of large groups of patients. A total of 27% of the staff reported that they already collaborated with (external) partners to this end.

#### 3.3.3 Context of VI team influences patient participation

According to the interviewees, several factors related to the context in which the patient participates can influence the level of patient participation. For example, the size of the VI team was mentioned as such a factor, since staff members believed that patients would not feel comfortable in large VI teams. Furthermore, the motivation of the medical lead to let patients participate or having a moderator who ensures that the patient is involved as an equal team member during the VI meetings were mentioned to facilitate patient participation. Additionally, digital meetings were experienced as challenging by patients and most patients preferred in‐person meetings with the VI team. Interviewees also indicated that the VI team discusses mainly medical subjects and not so much the patients' experience with care. Opinions differed on whether the VI team should change topics so that they would be more in line with what is relevant to patients.

#### 3.3.4 Resources required for patient participation

Limitations in resources were mainly discussed in the second round of interviews. Staff members indicated time and cost constraints as important challenges in patient participation. Only 38% of the staff who used a form of patient participation believed that patients' needs (such as financial, logistical and information needs) were accommodated. Furthermore, only half of the staff (46%) with experience in patient participation indicated that there was sufficient time allocated for patient participation.

Patients had varying opinions on whether they would like to receive financial compensation for their participation. Some patients indicated that financial compensation would make them feel like a more equal team member, while other patients indicated that they did not need any compensation for their participation in the VI team. The majority of patients indicated that they received sufficient support to participate in the VI teams (89%).

#### 3.3.5 Knowledge and skills in patient participation required from patients and staff

Interviewees, mainly from the first round, mentioned a list of characteristics and skills that patients should have to become a VI team member. Among these are being able to discuss in a constructive manner, being critical, being interested in healthcare, being motivated, being verbally strong, having a helicopter view and having basic knowledge of the healthcare system.

Patients mentioned that they have had some sort of introduction and explanation of the VI team before participation, but sometimes very limited. Additionally, patients indicated that they had previous experience with collective patient participation and that several patients were members of a patient organization and used the knowledge that they received from their organisation. The patients felt that they were equipped with the relevant skills, characteristics and knowledge required to be a VI team member. A reported challenge for patients to participate is the use of medical jargon in the VI team. All patients (100%) indicated that they had a clear understanding of the purpose of the VI team and that they had received sufficient information to participate in the team. Lastly, multiple staff members indicated in the interviews to struggle with patient participation, and 44% of the staff who had some experience in patient participation indicated that they would like to participate in a training on patient participation.

#### 3.3.6 Representativeness as a barrier for patient participation

The representativeness of patients who participate at the collective level was a much discussed topic in the interviews. Staff members expressed their concerns about letting patients participate in the VI team, since these patients would never be representative of the entire patient population. Furthermore, several staff members mentioned that it is important to include more than one patient, since ‘one is none’. This argument was mainly mentioned by staff members of VI teams with no form of patient participation. The majority of the staff (74%) believed that the involved patients reflected the targeted patient population of the VI team.

Patients stated that they did not feel like a representative patient, but that they tried to represent multiple perspectives. All patients (100%) indicated that they felt that they could represent the patients' perspectives, and 56% of the patients indicated that they felt more comfortable because they were not the only patient in the VI team.

#### 3.3.7 Relationship among VI team members influences patient participation

Some staff members stated in interview round one that it might be awkward for them to let a patient participate in VI meetings and that it would limit their ability to discuss topics that might be confronting for patients. Of the staff who had experience with a patient in their VI team, 80% indicated that this did not result in feeling restricted in their discussions.

Patients experienced some level of hierarchy in the VI team, but they did not feel restricted by this. Patients did indicate that it was important to them to keep their healthcare professional–patient relationship strictly separate from their relationship as team members. All patients (100%) stated that they felt able to freely express their opinions and that they felt like an equal team member. Therefore, the subject of hierarchy was not discussed in the second round of the interviews.

#### 3.3.8 Feedback and evaluation are important in patient participation

Patients reported that they sometimes missed feedback on what was done with their input. Moreover, both staff members and patients mentioned a lack of evaluation of patient participation within the VI team, resulting in little insight into the added value and effects of patient participation. However, in the questionnaire, both staff members with experience in patient participation (81%) and patients (73%) were positive about the feedback that patients received on their input.

Patients discussed several reasons why they were satisfied with their participation in the VI teams during the interviews, for example, since they felt taken seriously, of added value, and comfortable in the VI team. Yet, negative experiences with patient participation were also mentioned, for example, patients felt tokenistic in the beginning, or staff members felt that the input delivered by patients was not very helpful or insightful. Overall, the majority of patients (87%) felt that their participation was a good use of their time and were satisfied with their role in the VI team. On the contrary, only 41% of the staff with experience in patient participation were satisfied with this patient participation in their VI team.

#### 3.3.9 Impact of patient participation

Staff members indicated that they believe in the added value of patient participation, since patients have a unique expertise that would be missed without patient participation. Both patients and staff members stated that patient participation has increased over time and that steps are being taken to improve patient participation even more within the VI teams. However, other staff members believed that patients should participate on another level than the VI team, because being a VI team member is too burdensome for the patient. This was mentioned several times in round one as a reason for not letting a patient participate in the VI team.

Most patients believed that their input was taken into consideration (93%). In addition, staff members of teams with patient participation indicated that the input of patients was taken seriously (78%), but only half of the staff also believed that the input of patients had influence on the outcomes of the VI team (51%). The majority of the staff members (90%) with experience in patient participation believed that it had added value.

Lastly, in addition to the impact of patient participation on the VI team in general, it can have an impact on the individuals in the VI team. Patients indicated that because of their participation, they were better informed about their medical condition and/or the healthcare system (73%).

## 4 DISCUSSION

In this study, we aimed to evaluate patient participation within VI teams. The results showed that currently only half of the 76 included VI teams use a form of patient participation. Nine themes regarding the experiences with patient participation in the VI teams were identified in the interviews, of which we would like to highlight four interesting observations. First, the majority of VI teams lacked a clear strategy and goal for patient participation, which may explain why half of the VI teams had no form of patient participation. Second, we found that specific knowledge and skills are required for patients to participate in a VI team. This leads to the inclusion of patients with these specific skills, which in turn leads to concerns regarding the representativeness of participating patients. Third, patients indicated to have an equal and informal relationship with the rest of the VI team and did not experience ‘hierarchy’ as a challenge for participation. Fourth, patients mentioned in interviews a lack of feedback on their input, but this was not found in the quantitative results. Meanwhile, staff members indicated in the questionnaire that the input of patients often did not influence the outcomes of the VI trajectory. We discuss these four observations in greater detail below.

This study describes many challenges for patient participation, which may be related to the lack of strategy for patient participation that staff members reported. Having a clear strategy, including clarity on the role for the patient, recruitment of patients, and funding and time investments, could provide a solution for the experienced difficulties in the implementation of patient participation.^16^, ^25^, ^26^, ^27^ Additionally, several practical aspects were mentioned that could stimulate patient participation in VI teams but are currently absent, for example, ‘having a trained moderator to guide the team meetings’. This importance of a moderator trained in patient participation techniques has been stipulated before in the literature.^16^, ^28^, ^29^ Furthermore, multiple methods for patient participation can be used by the VI teams. Having a patient as a team member is often seen as the highest level of patient participation, and therefore the best possible method. This hierarchy is implied by the frequently used ‘ladder of participation’ of Arnstein.^30^ However, as Carman et al. also discuss in their framework, the ‘highest’ level of participation on the continuum of engagement is not always the best level, but it depends on the question, aim, or project.^6^ This was also pointed out by the VI team members, but they indicated to struggle with the selection of suitable patient participation methods for their VI team. Therefore, the VI teams would benefit from guidelines on patient participation, which include the different methods and levels of patient participation. Existing frameworks such as frameworks for patient participation in research^31^, ^32^ and the framework of Carman et al.^6^ could be used to develop such guidelines.

Our results show the importance of paying attention to knowledge and skills of both patients and staff regarding patient participation. In the interviews, staff members and patients indicated several skills and a certain level of knowledge that a patient should have when participating in a VI team. All patients in our study indicated that they possessed these skills. However, staff members also discussed their concern regarding the required skills for participating patients, since patients with these skills might not be representative for the entire patient population. They indicated that other methods of patient participation, such as focus groups, could help to gain insight into the perspectives of a larger group of patients. Previous research has recommended combining the two methods, so having both patient representatives as well as using other methods such as patient questionnaires, to learn more about the perspectives of a large group of patients.^33^ Furthermore, in the literature, there are different opinions on the level of professionalism that is required from patients to participate at the collective level. Some studies stipulate the importance of training the patients,^9^, ^34^, ^35^, ^36^, ^37^, ^38^ while others criticize this need of training, since this would lead to ‘proto‐professionalism’ of patients participating in such projects and minimizes the representativeness of patients.^39^ Training of staff in patient participation is less controversial, and previous studies have shown positive effects of such training.^34^, ^35^, ^40^, ^41^ However, only half of the staff in our study indicated that they would like to be trained in patient participation.

Hierarchy is an often mentioned barrier for patient participation,^42^, ^43^ and while our results show that patients experienced some level of hierarchy, patients also stated that this did not make them feel restricted to express their opinion freely. Almost half of the patients reported that having another patient as a team member did not result in them feeling more comfortable in the VI team. This contradicts a recommendation of a review on patient participation, where it was suggested to have an equal or higher number of patients compared to staff in a project team to strengthen patient voices.^16^ This contradiction might be explained by the fact that there is a high level of patient empowerment in Dutch healthcare,^44^ likely resulting in Dutch patients feeling more equal to their healthcare professionals than patients from other countries. Patients did indicate that it is important to maintain their healthcare professional–patient relationship strictly separate from their relationship as VI team members, but that it is possible to participate in a VI team with their own doctor or nurse. This is only partially in line with previous research, where they found that patients should not have a prior relationship with the healthcare professionals in the same panel, since patients would not feel comfortable with that.^28^ Morever, not only patients but also healthcare professionals seem to find it challenging to shift their doctor–patient relationship to a ‘colleague’ relationship.^45^ During the first round of interviews, several staff members mentioned that having a patient in the VI team member would restrict them from expressing their opinion freely, while results from the questionnaire contradicted this finding. Therefore, we recommend that patients and staff members discuss this topic (‘hierarchy’) before becoming team members, since both parties seem to struggle with it on their own.

Patients believed their input was taken seriously and into consideration, but during the interviews, several patients indicated that they were not sure how their input was used in practice. Meanwhile, staff members who had experience with patient participation indicated that the input of patients had a limited effect in practice. These results indicate that, also in teams where patients were equal team members, often no full partnership was reached (yet). The lack of identified impact of patient participation might be related to the lack of a clear goal for patient participation. Without a clear goal, it is challenging to determine the effects, since it has not been predetermined how and what to measure as effects. This shortcoming in evaluation and feedback on the impact of patient participation has been addressed before in literature. This problem could be tackled by having a coordinator for patient participation with assigned time for evaluation.^46^ Lastly, previous research identified a lack of formal tools to evaluate the effect of collective patient participation.^47^ A strategy for patient participation should therefore also include an evaluation plan for the effects of patient participation.

Some limitations deserve consideration in light of these results. First, only fifteen patients completed the questionnaire and they were all very satisfied with their participation. We are unsure if our sample was representative, since most patients mentioned during the interviews that they would leave the VI team if they were dissatisfied. Since the questionnaire was only sent out to the patients who were still part of the VI teams, it would also be interesting for future studies to include patients who were less satisfied, for example, by inviting patients who have ceased their participation to the VI team. Second, since the patients included in our study were all highly educated, we were not able to gain new insights into strategies for enhancing patient participation of patients with more diverse characteristics. Patient participation can be achieved with other (less structural) methods, such as organizing a focus group to gain insight into a specific subject. It would be interesting to evaluate patient participation from the perspectives of these patients as well and to evaluate whether these methods would stimulate patient participation from patients with diverse characteristics. Third, we were unable to calculate a response rate, since we asked the project leaders to forward the questionnaire to ‘their’ VI team members and we are unsure who they invited. Fourth, several VI team members of the same VI team gave conflicting answers regarding the method of patient participation used by their VI team. This indicates that not all VI team members are completely up to date on patient participation in their teams, which could have resulted in unreliable answers to the questionnaire.

## 5 CONCLUSION

These findings provide both qualitative and quantitative insights into the current state of and experiences with patient participation in multidisciplinary VI teams. The results imply that patient participation is not (yet) fully implemented within the current practice of the VI teams. Therefore, we believe that VI team members would benefit from guidelines on how to include a representative group of patients, how to train both patients and staff in patient participation, which methods to use, how to evaluate the impact of patient participation, and how to provide feedback to participating patients. These guidelines could be based on the findings of this study and existing frameworks and theories on patient participation. Future studies should aim to formulate and implement such guidelines to improve patient participation within VBHC initiatives.

## AUTHOR CONTRIBUTIONS

**Henrike J. Westerink**: Conceptualization; methodology; formal analysis; investigation; writing—original draft; project administration. **Mirjam M. Garvelink**: Conceptualization; funding acquisition; methodology; writing—review and editing; formal analysis; supervision. **Cornelia F. van Uden‐Kraan:** Conceptualization, methodology; formal analysis; writing—review & editing; supervision; funding acquisition. **Ouisam Zouitni**: Conceptualization; methodology; formal analysis; writing—review and editing. **Hans A. J. Bart**: Conceptualization; methodology; formal analysis; writing—review and editing. **Philip J. van der Wees**: Conceptualization; methodology; formal analysis; supervision; writing—review and editing; funding acquisition. **Paul B. van der Nat**: Conceptualization; funding acquisition; methodology; writing—review and editing; formal analysis; supervision.

## CONFLICT OF INTEREST STATEMENT

The authors declare no conflict of interest.

## ETHICS STATEMENT

This research proposal has been approved by the Medical Ethics Committee (Santeon Beheercommissie SDB2021‐014 and the Medical Ethical Commission‐Utrecht W22.212). All participating hospitals also received approval from their scientific committee for local feasibility. Participants gave informed consent to use their data for the study.

## SANTEON PATIENT PARTICIPATION STUDY GROUP

The collaborators of the Santeon Patient Participation Study Group are as follows: Annette W. G. van der Velden, Martini Hospital, Groningen, the Netherlands. Sander Koëter, Canisius Wilhelmina Hospital, Nijmegen, the Netherlands. Willem J. W. Bos, St. Antonius Hospital, Utrecht/Nieuwegein, the Netherlands. Diederik H. R. Kempen, OLVG, Amsterdam, the Netherlands. Angelique E. A. M. Weel, Maasstad Hospital, Rotterdam, the Netherlands. Eino B. van Duyn, Medisch Spectrum Twente, Enschede, the Netherlands. Pepijn H. van der Voort, Catharina Hospital, Eindhoven, the Netherlands.

## Supporting information

Supporting information.

Supporting information.

Supporting information.

## Data Availability

The data that support the findings of this study are available on request from the corresponding author. The data are not publicly available due to privacy or ethical restrictions.
