# Supplementary material for: Evaluating patient participation in value‐based healthcare: Current state and lessons learned
Source: Health Expect. 2024 Jan 18;27(1):e13945. doi: 10.1111/hex.13945 (PMC10797212; doi:10.1111/hex.13945)
Supplement: Supplementary file 1 — Supporting information. [file HEX-27-e13945-s001.docx]

# Appendix A: Interview guides

### Interview Round 1:

**Interview questions patients:**

1. How do patients participate in your Value Improvement (VI) team?
2. Can you tell me something about your role in the VI team?
3. Who invited you to become a VI team member?
4. Why did you chose to become a VI team member?
5. Do you think it is important that a patient is part of the VI team? Why?
6. What do you want to accomplish by being a VI team member?
7. What do you think that the effects are of a patient being a VI team member?
8. What do you need to participate properly in the VI team?
9. What do you expect from other VI team members?
10. Do you have an example of a moment when you felt that the patient participation was successful? Why was that?
11. Do you have an example of a moment when you felt that the patient participation was unsuccessful? Why was that?
12. Having a patient as VI team member is one method for patient participation. Do you have other ideas of how to involve patients in quality improvement projects/programs in healthcare?

**Interview questions staff:**

1. How are patients being involved in your VI team? Do you have a patient as VI team member? Are patients also being involved in another way?
2. Do you think that patient participation is of added value in the VI team? Why?
3. Do you think it is important to have a patient as VI team member? Why (not)?
4. What do you want to accomplish by conducting patient participation?
5. What do you expect from the patient as VI team member? What is the role of the patient?
6. What can you do to support the patient in the VI team? What do you need to support the patient within the VI team?
7. Do you have an example of a moment when you felt that the patient participation was successful? Why was that?
8. Do you have an example of a moment when you felt that the patient participation was unsuccessful? Why was that?
9. Having a patient as VI team member is one method for patient participation. Do you have other ideas of how to involve patients in quality improvements in healthcare?

### Interview Round 2:

1. How are patients being involved in your VI team?
2. Would you like to explain a few answers you gave in the questionnaire? (this question was individually prepared for every interview)
3. How effective do you think that the patient participation in your VI team is?
4. What is going well in patient participation in your VI team?
5. What can be improved in patient participation in your VI team?
6. What is the biggest barrier in patient participation in your VI team?
7. How would you solve this barrier?
8. What do you need from your team or organisation to solve this barrier?
9. Is everyone in your VI team positive about patient participation?
10. Would you like to use other methods for patient participation? If yes, why and which methods?
