# Supplementary material for: Evaluating patient participation in value‐based healthcare: Current state and lessons learned
Source: Health Expect. 2024 Jan 18;27(1):e13945. doi: 10.1111/hex.13945 (PMC10797212; doi:10.1111/hex.13945)
Supplement: Supplementary file 2 — Supporting information. [file HEX-27-e13945-s003.docx]

# Appendix B

## Questionnaire results of patients:

| **Question number** | **Question** | **N score 0** | **N score 1** | **N score 2** | **N score 3** | **N score 4** | **N score NA** | **N respondents** | **% agree** |
| --- | --- | --- | --- | --- | --- | --- | --- | --- | --- |
| 1 | I have a clear understanding of the purpose of the VI team. | 0 | 0 | 0 | 6 | 9 |  | 15 | 100% |
| 2 | The supports I need to participate in the VI team are available (e.g., travel, childcare) | 0 | 0 | 1 | 4 | 4 |  | 9 | 89% |
| 3 | I have enough information to be able to carry out my role. | 0 | 0 | 0 | 7 | 8 |  | 15 | 100% |
| 4 | I feel like I can represent the patient perspective | 0 | 0 | 0 | 6 | 9 |  | 15 | 100% |
| 5 | I am able to express my view freely. | 0 | 0 | 0 | 1 | 14 |  | 15 | 100% |
| 6 | I feel that my views are heard. | 0 | 0 | 1 | 2 | 12 |  | 15 | 93% |
| 7 | A wide range of views on discussion topics is shared. | 0 | 0 | 3 | 7 | 5 |  | 15 | 80% |
| 8 | The individuals participating in the VI team represent a broad range of perspectives. | 0 | 0 | 2 | 7 | 6 |  | 15 | 87% |
| 9 | I feel more comfortable, because I am not the only patient in the VI team. | 0 | 0 | 4 | 4 | 1 |  | 9 | 56% |
| 10 | I feel like an equal VI team member | 0 | 0 | 0 | 7 | 8 |  | 15 | 100% |
| 11 | I think that the hospital is achieving its stated objectives for the VI team. | 0 | 0 | 5 | 9 | 1 |  | 15 | 67% |
| 12 | I am confident that the VI team takes my provided feedback into consideration | 0 | 0 | 1 | 8 | 6 |  | 15 | 93% |
| 13 | I think that the work of the VI team makes a difference to the work of the hospital | 0 | 1 | 1 | 10 | 3 |  | 15 | 87% |
| 14 | I get sufficient feedback on how my input is used | 0 | 1 | 3 | 8 | 3 |  | 15 | 73% |
| 15 | As a result of my participation in the VI team, I am better informed about my health condition and/or the healthcare system. | 0 | 3 | 1 | 7 | 4 |  | 15 | 73% |
| 16 | Overall, I am satisfied with the patient participation in this project. | 0 | 0 | 2 | 7 | 6 |  | 15 | 87% |
| 17 | This initiative for patient participation is a good use of my time. | 0 | 0 | 2 | 5 | 8 |  | 15 | 87% |

## Questionnaire results of staff:

| **Question number** | **Question** | **N score 0** | **N score 1** | **N score 2** | **N score 3** | **N score 4** | **N score NA** | **N respondents** | **% agree** |
| --- | --- | --- | --- | --- | --- | --- | --- | --- | --- |
| 1 | There was a clear statement of the objectives for the engagement. | 4 | 30 | 41 | 33 | 14 | 5 | 127 | 39% |
| 2 | The VI team has a clear strategy to recruit those most affected by the outputs of this project (e.g. relevant lived experience, socio-demographic or geographic communities) | 10 | 38 | 37 | 27 | 7 | 7 | 126 | 29% |
| 3 | The VI team has a clear strategy to actively engage patients (if no form of patient participation was used, the questionnaire ended here) | 4 | 38 | 42 | 32 | 6 | 6 | 128 | 31% |
| 4 | The perspectives of those who will be most affected by the outputs of this project were reflected through those who participated in the engagement. | 2 | 4 | 15 | 43 | 16 | 1 | 81 | 74% |
| 5 | The financial, logistical and information needs of participants (e.g., travel, dietary, interpretive, childcare, etc.) were accommodated. | 3 | 15 | 17 | 19 | 2 | 1 | 57 | 38% |
| 6 | Adequate time was allocated to plan and implement the engagement component. | 2 | 7 | 29 | 30 | 3 | 1 | 72 | 46% |
| 7 | The goals for the engagement component were shared with participants. | 1 | 6 | 11 | 45 | 9 | 1 | 73 | 75% |
| 8 | Participants were told how the input from the engagement component would be used by the organization. | 0 | 2 | 12 | 49 | 11 | 1 | 75 | 81% |
| 9 | I can speak freely with the patient at the table. | 1 | 2 | 4 | 22 | 6 | 1 | 36 | 80% |
| 10 | We worked with other (patient)organizations as part of the engagement component of this project. (0=no, 1=yes) | 66 | 25 |  |  |  |  | 91 | 27% |
| 11 | If Q10=yes: We were able to identify shared goals with our partners through this process. | 0 | 1 | 6 | 14 | 1 |  | 22 | 68% |
| 12 | If Q10=yes: Do you plan to collaborate with these partners again in the future? (0=no, 1=yes) | 2 | 23 |  |  |  |  | 25 | 92% |
| 13 | Overall, I was satisfied with the engagement component of this project. | 3 | 22 | 26 | 31 | 4 |  | 86 | 41% |
| 14 | The engagement component added value to the project it supported. | 1 | 1 | 6 | 52 | 21 |  | 81 | 90% |
| 15 | As a result of my involvement in the engagement component associated with this project, I will be comfortable leading future engagement activities. | 0 | 9 | 22 | 42 | 4 | 1 | 78 | 60% |
| 16 | I would like to participate in a training on patient participation (0=no, 1=yes) | 51 | 40 |  |  |  |  | 91 | 44% |
| 17 | The output generated from the engagement component associated with this project were taken seriously by those in a position to act on it | 0 | 3 | 14 | 43 | 18 |  | 78 | 78% |
| 18 | The output generated from the engagement component associated with this project influenced the project’s outcome | 0 | 7 | 31 | 34 | 5 | 1 | 78 | 51% |
