# Supplementary material for: Evaluating patient participation in value‐based healthcare: Current state and lessons learned
Source: Health Expect. 2024 Jan 18;27(1):e13945. doi: 10.1111/hex.13945 (PMC10797212; doi:10.1111/hex.13945)
Supplement: Supplementary file 3 — Supporting information. [file HEX-27-e13945-s002.docx]

# Appendix C

## Step-by-step guide of data analysis:

1. The semi-structured interviews of round one and two were recorded and transcribed verbatim.
2. The transcripts were sent to the interviewees for a member check, and no changes were made by the interviewees.
3. The transcripts were loaded into Atlas.ti. Since the interview guides of round one and two were not very different from each other, we analyzed the interviews of both rounds in the same manner.
4. The first five interviews were independently coded with an inductive approach by both HJW (first author) and OW (research intern).
5. HJW and OW discussed the codes of the first five interviews and reached consensus on the open codes.
6. The next five interviews were open coded independently by HJW and OW and discussed in a consensus meeting.
7. This process was continued until all interviews were coded by the two researchers and consensus was reached on all open codes.
8. All codes were thematically grouped in an session with HJW and OW by printing all the codes and grouping them on different post-its. They reached consensus on the identified subthemes and wrote these subthemes on the post-its. These post-it’s were then placed into theme’s by grouping them and adding them to envelops with the theme’s written on it.
9. These themes and subthemes were then discussed with the co-authors and consensus was reached.
10. These results were then shared with the interviewees. No comments were made that would lead to changing the themes.
11. The data of the questionnaire was analyzed using Excel. The percentages of respondents who (totally) agreed with the individual questions were calculated.
12. HJW then matched the questions to the identified themes. This matching was then discussed in a meeting with the co-authors. This method allows to visualize both the qualitative data and quantitative data in one overview figure (triangulation).
13. The quantitative results were then placed in a schematic overview of all interview themes and subthemes.
